# 2,4-Dimethyl-*N*-phenyl­benzene­sulfonamide

**DOI:** 10.1107/S160053680900573X

**Published:** 2009-02-21

**Authors:** B. Thimme Gowda, Sabine Foro, P. G. Nirmala, K. S. Babitha, Hartmut Fuess

**Affiliations:** a Department of Chemistry, Mangalore University, Mangalagangotri 574 199, Mangalore, India; b Institute of Materials Science, Darmstadt University of Technology, Petersenstrasse 23, D-64287 Darmstadt, Germany

## Abstract

The asymmetric unit of the crystal structure of the title compound, C_14_H_15_NO_2_S, contains two mol­ecules. The conformations of the N—C bonds in the C—SO_2_—NH—C segments of the structure have *trans* and *gauche* torsion angles with the S=O bonds. Furthermore, the torsion angles of the C—SO_2_—NH—C groups in the two mol­ecules are 46.1 (3) (glide image of mol­ecule 1) and 47.7 (3)° (mol­ecule 2). The *ortho*-methyl groups in the sulfonyl benzene ring are oriented away from the S=O bonds. The two benzene rings are tilted relative to each other by 67.5 (1) and 72.9 (1)° in the two mol­ecules. N—H⋯O and C—H⋯O hydrogen bonds pack the mol­ecules into one-dimensional chains in different directions, resulting in a two-dimensional network.

## Related literature

For related structures, see: Gelbrich *et al.* (2007 ▶); Gowda *et al.* (2008**a* ▶,*b* ▶,c* ▶); Perlovich *et al.* (2006 ▶).
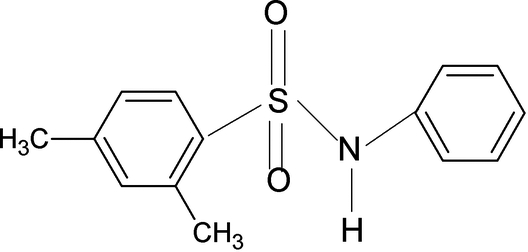


## Experimental

### 

#### Crystal data

C_14_H_15_NO_2_S
*M* *_r_* = 261.33
Orthorhombic, 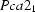

*a* = 19.113 (3) Å
*b* = 8.9290 (8) Å
*c* = 15.781 (1) Å
*V* = 2693.2 (5) Å^3^
*Z* = 8
Cu *K*α radiation
μ = 2.09 mm^−1^
*T* = 299 K
0.50 × 0.43 × 0.25 mm

#### Data collection

Enraf–Nonius CAD-4 diffractometer
Absorption correction: ψ scan (North *et al.*, 1968 ▶) *T*_min_ = 0.401, *T*_max_ = 0.593
4916 measured reflections
2505 independent reflections
2421 reflections with *I* > 2σ(*I*)
*R*_int_ = 0.050
3 standard reflections frequency: 120 min intensity decay: 1.0%

#### Refinement

*R*[*F* ^2^ > 2σ(*F* ^2^)] = 0.034
*wR*(*F* ^2^) = 0.090
*S* = 1.09
2505 reflections
330 parameters
7 restraints
H-atom parameters constrained
Δρ_max_ = 0.26 e Å^−3^
Δρ_min_ = −0.23 e Å^−3^
Absolute structure: Flack (1983 ▶), no Friedel pairs
Flack parameter: 0.008 (17)

### 

Data collection: *CAD-4-PC* (Enraf–Nonius, 1996 ▶); cell refinement: *CAD-4-PC*; data reduction: *REDU4* (Stoe & Cie, 1987 ▶); program(s) used to solve structure: *SHELXS97* (Sheldrick, 2008 ▶); program(s) used to refine structure: *SHELXL97* (Sheldrick, 2008 ▶); molecular graphics: *PLATON* (Spek, 2009 ▶); software used to prepare material for publication: *SHELXL97*.

## Supplementary Material

Crystal structure: contains datablocks I, global. DOI: 10.1107/S160053680900573X/kj2115sup1.cif

Structure factors: contains datablocks I. DOI: 10.1107/S160053680900573X/kj2115Isup2.hkl

Additional supplementary materials:  crystallographic information; 3D view; checkCIF report

## Figures and Tables

**Table 1:** Hydrogen-bond geometry (Å, °)

*D*—H⋯*A*	*D*—H	H⋯*A*	*D*⋯*A*	*D*—H⋯*A*
N1—H1*N*⋯O3^i^	0.86	2.41	3.164 (3)	147
N2—H2*N*⋯O1^ii^	0.86	2.22	3.056 (3)	164
C11—H11⋯O2^iii^	0.93	2.50	3.227 (4)	135
C23—H23⋯O4^iii^	0.93	2.50	3.316 (4)	147

Symmetry codes: (i) 
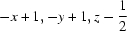
; (ii) 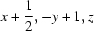; (iii) 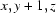.

**Fig. 1.**
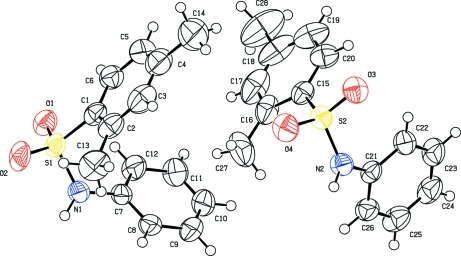
Molecular structure of the title compound, showing the atom labeling scheme. The displacement ellipsoids are drawn at the 50% probability level. The H atoms are represented as small spheres of arbitrary radii.

**Fig. 2.**
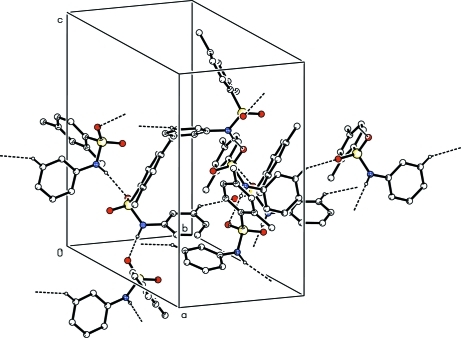
Molecular packing of the title compound with hydrogen bonding shown as dashed lines. H-atoms not involved in hydrogen bonding have been omitted.

## supplementary crystallographic information

### Comment

As part of a study of the substituent effects on the crystal structures of *N*-(aryl)-arylsulfonamides (Gowda *et al.*, 2008*a, b, c*), in the present work, the structure of *N*-(phenyl)-2,4-dimethylbenzenesulfonamide has been determined . The asymmetric unit contains 2 molecules (Fig. 1). The conformations of the N—C bonds in the C—SO_2_—NH—C segments of the structure have  "trans" torsions and "gauche" torsions with the S═O bonds. Further, the torsion angles of the C—SO_2_—NH—C groups in the two molecules are 46.1 (3)° (glide image of molecule 1) and 47.7 (3)° (molecule 2). The *ortho*-methyl groups in the sulfonyl benzene rings orient themselves away from the S═O bonds, but in the direction of N—H bonds. The two benzene rings in the title compound are tilted relative to each other by 67.5 (1)° in the molecule 1 and 72.9 (1)° in molecule 2. The other bond parameters in the title compound are similar to those observed in *N*-(2,6-dimethylphenyl)-benzenesulfonamide (Gowda *et al.*, 2008*a*), *N*-(2-methylphenyl)-benzenesulfonamide (Gowda *et al.*, 2008*b*)) and other aryl sulfonamides (Perlovich *et al.*, 2006; Gelbrich *et al.*, 2007;  Gowda *et al.*, 2008*c*). The N-H···O hydrogen bonds pack the molecules into a 1D chain in the direction of *c*- axis, while C-H···O hydrogen bonds pack them into a 1D chain in the direction of *b*-axis, resulting in a 2D network (Table 1, Fig. 2).

### Experimental

A solution of 1,3-xylene (10 ml) in chloroform (40 ml) was treated dropwise with chlorosulfonic acid (25 ml) at 273K. After the initial evolution of hydrogen chloride subsided, the reaction mixture was brought to room temperature and poured into crushed ice in a beaker. The chloroform layer was separated, washed with cold water and allowed to evaporate slowly. The residual 2,4-dimethylbenzenesulfonylchloride was treated with aniline in the stoichiometric ratio and boiled for ten minutes. The reaction mixture was then cooled to room temperature and added to ice cold water (100 ml). The resultant solid *N*-(phenyl)-2,4-dimethylbenzenesulfonamide was filtered under suction and washed thoroughly with cold water. It was then recrystallized to constant melting point from dilute ethanol. The purity of the compound was checked and characterized by recording its infrared and NMR spectra. The single crystals used in X-ray diffraction studies were grown in ethanolic solution by slow evaporation at room temperature.

### Refinement

The H atoms were positioned with idealized geometry using a riding model with C—H = 0.93–0.96 Å, N—H = 0.86 Å, and were refined with isotropic displacement parameters (set to 1.2 times of the *U*_eq_ of the parent atom). The U^ij^ components of C28 were restrained to approximate isotropic behavior.

### Crystal data

**Table:**

C_14_H_15_NO_2_S	*F*(000) = 1104
*M**_r_* = 261.33	*D*_x_ = 1.289 Mg m^−^^3^
Orthorhombic, *P**c**a*2_1_	Cu *K*α radiation, λ = 1.54180 Å
Hall symbol: P 2c -2ac	Cell parameters from 25 reflections
*a* = 19.113 (3) Å	θ = 4.6–19.0°
*b* = 8.9290 (8) Å	µ = 2.09 mm^−^^1^
*c* = 15.781 (1) Å	*T* = 299 K
*V* = 2693.2 (5) Å^3^	Prism, colourless
*Z* = 8	0.50 × 0.43 × 0.25 mm

### Data collection

**Table:**

Enraf–Nonius CAD-4 diffractometer	2421 reflections with *I* > 2σ(*I*)
Radiation source: fine-focus sealed tube	*R*_int_ = 0.050
graphite	θ_max_ = 66.9°, θ_min_ = 4.6°
ω/2θ scans	*h* = −22→22
Absorption correction: ψ scan (North *et al.*, 1968)	*k* = −10→0
*T*_min_ = 0.401, *T*_max_ = 0.593	*l* = −18→0
4916 measured reflections	3 standard reflections every 120 min
2505 independent reflections	intensity decay: 1.0%

### Refinement

**Table:**

Refinement on *F*^2^	Hydrogen site location: inferred from neighbouring sites
Least-squares matrix: full	H-atom parameters constrained
*R*[*F*^2^ > 2σ(*F*^2^)] = 0.034	*w* = 1/[σ^2^(*F*_o_^2^) + (0.0565*P*)^2^ + 0.1061*P*] where *P* = (*F*_o_^2^ + 2*F*_c_^2^)/3
*wR*(*F*^2^) = 0.090	(Δ/σ)_max_ = 0.012
*S* = 1.09	Δρ_max_ = 0.26 e Å^−^^3^
2505 reflections	Δρ_min_ = −0.23 e Å^−^^3^
330 parameters	Extinction correction: *SHELXL97* (Sheldrick, 2008), Fc^*^=kFc[1+0.001xFc^2^λ^3^/sin(2θ)]^-1/4^
7 restraints	Extinction coefficient: 0.0032 (3)
Primary atom site location: structure-invariant direct methods	Absolute structure: Flack (1983), no Friedel pairs
Secondary atom site location: difference Fourier map	Flack parameter: 0.008 (17)

### Special details

**Table:**

Geometry. All e.s.d.'s (except the e.s.d. in the dihedral angle between two l.s. planes) are estimated using the full covariance matrix. The cell e.s.d.'s are taken into account individually in the estimation of e.s.d.'s in distances, angles and torsion angles; correlations between e.s.d.'s in cell parameters are only used when they are defined by crystal symmetry. An approximate (isotropic) treatment of cell e.s.d.'s is used for estimating e.s.d.'s involving l.s. planes.
Refinement. Refinement of *F*^2^ against ALL reflections. The weighted *R*-factor *wR* and goodness of fit *S* are based on *F*^2^, conventional *R*-factors *R* are based on *F*, with *F* set to zero for negative *F*^2^. The threshold expression of *F*^2^ > σ(*F*^2^) is used only for calculating *R*-factors(gt) *etc*. and is not relevant to the choice of reflections for refinement. *R*-factors based on *F*^2^ are statistically about twice as large as those based on *F*, and *R*- factors based on ALL data will be even larger.

### Fractional atomic coordinates and isotropic or equivalent isotropic displacement parameters (Å^2^)

**Table:**

	*x*	*y*	*z*	*U*_iso_*/*U*_eq_	
S1	0.25250 (4)	0.26684 (7)	0.22911 (5)	0.05031 (19)	
O1	0.18269 (11)	0.3237 (2)	0.23659 (15)	0.0594 (5)	
O2	0.26004 (13)	0.1099 (2)	0.21441 (18)	0.0702 (7)	
N1	0.29100 (14)	0.3475 (3)	0.14888 (15)	0.0544 (5)	
H1N	0.3002	0.2948	0.1046	0.065*	
C1	0.29873 (15)	0.3238 (3)	0.32001 (19)	0.0520 (6)	
C2	0.36811 (17)	0.2791 (4)	0.3346 (2)	0.0603 (7)	
C3	0.39906 (19)	0.3302 (5)	0.4076 (3)	0.0763 (10)	
H3	0.4448	0.3004	0.4188	0.092*	
C4	0.36652 (19)	0.4232 (5)	0.4657 (2)	0.0767 (9)	
C5	0.2984 (2)	0.4650 (4)	0.4490 (2)	0.0737 (9)	
H5	0.2748	0.5266	0.4869	0.088*	
C6	0.26517 (16)	0.4164 (3)	0.3769 (2)	0.0587 (6)	
H6	0.2194	0.4464	0.3663	0.070*	
C7	0.31002 (15)	0.5019 (3)	0.14938 (15)	0.0472 (5)	
C8	0.37518 (17)	0.5411 (3)	0.11944 (17)	0.0559 (6)	
H8	0.4067	0.4679	0.1022	0.067*	
C9	0.3931 (2)	0.6918 (4)	0.1154 (2)	0.0679 (9)	
H9	0.4363	0.7200	0.0935	0.081*	
C10	0.3472 (2)	0.7996 (3)	0.1438 (2)	0.0693 (9)	
H10	0.3596	0.9003	0.1414	0.083*	
C11	0.2844 (2)	0.7591 (3)	0.1749 (3)	0.0706 (8)	
H11	0.2539	0.8323	0.1947	0.085*	
C12	0.26465 (19)	0.6103 (3)	0.1777 (2)	0.0633 (7)	
H12	0.2209	0.5836	0.1987	0.076*	
C13	0.4085 (2)	0.1767 (5)	0.2756 (3)	0.0844 (12)	
H13A	0.4131	0.2237	0.2211	0.101*	
H13B	0.3838	0.0837	0.2695	0.101*	
H13C	0.4541	0.1579	0.2987	0.101*	
C14	0.4032 (3)	0.4784 (9)	0.5438 (3)	0.1129 (18)	
H14A	0.4524	0.4585	0.5392	0.135*	
H14B	0.3848	0.4278	0.5927	0.135*	
H14C	0.3959	0.5843	0.5497	0.135*	
S2	0.58393 (3)	0.79647 (7)	0.45792 (4)	0.04598 (18)	
O3	0.61521 (12)	0.8591 (3)	0.53213 (13)	0.0605 (5)	
O4	0.59126 (13)	0.6383 (2)	0.44450 (15)	0.0665 (6)	
N2	0.61782 (13)	0.8727 (3)	0.37433 (15)	0.0509 (5)	
H2N	0.6431	0.8164	0.3424	0.061*	
C15	0.49526 (13)	0.8489 (3)	0.45929 (17)	0.0496 (5)	
C16	0.44843 (19)	0.7964 (4)	0.3987 (2)	0.0673 (9)	
C17	0.3784 (2)	0.8473 (6)	0.4075 (3)	0.0906 (14)	
H17	0.3454	0.8140	0.3684	0.109*	
C18	0.35654 (19)	0.9422 (6)	0.4700 (3)	0.0949 (15)	
C19	0.4042 (2)	0.9912 (6)	0.5270 (3)	0.0854 (12)	
H19	0.3904	1.0567	0.5697	0.102*	
C20	0.47306 (17)	0.9454 (4)	0.52259 (19)	0.0634 (8)	
H20	0.5050	0.9797	0.5626	0.076*	
C21	0.60945 (12)	1.0244 (3)	0.34916 (17)	0.0451 (5)	
C22	0.60950 (17)	1.1394 (3)	0.4086 (2)	0.0569 (6)	
H22	0.6138	1.1185	0.4660	0.068*	
C23	0.6030 (2)	1.2854 (4)	0.3806 (3)	0.0678 (9)	
H23	0.6019	1.3629	0.4199	0.081*	
C24	0.5981 (2)	1.3182 (4)	0.2961 (3)	0.0686 (9)	
H24	0.5945	1.4172	0.2782	0.082*	
C25	0.5986 (2)	1.2031 (4)	0.2377 (3)	0.0692 (8)	
H25	0.5956	1.2244	0.1801	0.083*	
C26	0.60355 (16)	1.0569 (3)	0.26450 (18)	0.0555 (7)	
H26	0.6029	0.9797	0.2250	0.067*	
C27	0.4682 (3)	0.6917 (6)	0.3282 (3)	0.1016 (16)	
H27A	0.4895	0.6034	0.3516	0.122*	
H27B	0.5008	0.7405	0.2910	0.122*	
H27C	0.4271	0.6641	0.2970	0.122*	
C28	0.2808 (2)	0.9939 (10)	0.4738 (4)	0.136 (2)	
H28A	0.2542	0.9255	0.5079	0.164*	
H28B	0.2617	0.9966	0.4175	0.164*	
H28C	0.2787	1.0922	0.4983	0.164*	

### Atomic displacement parameters (Å^2^)

**Table:**

	*U*^11^	*U*^22^	*U*^33^	*U*^12^	*U*^13^	*U*^23^
S1	0.0539 (3)	0.0320 (3)	0.0650 (4)	−0.0034 (3)	0.0112 (3)	0.0002 (3)
O1	0.0510 (9)	0.0539 (10)	0.0733 (12)	−0.0010 (9)	0.0070 (10)	0.0024 (11)
O2	0.0789 (14)	0.0290 (9)	0.1028 (18)	−0.0078 (10)	0.0143 (12)	−0.0020 (11)
N1	0.0726 (13)	0.0345 (10)	0.0562 (11)	−0.0029 (11)	0.0167 (11)	−0.0072 (9)
C1	0.0531 (13)	0.0439 (13)	0.0591 (14)	−0.0029 (12)	0.0083 (12)	0.0089 (11)
C2	0.0536 (14)	0.0536 (15)	0.0736 (18)	0.0048 (12)	0.0098 (13)	0.0140 (14)
C3	0.0591 (16)	0.086 (3)	0.084 (2)	−0.0040 (18)	−0.0014 (16)	0.021 (2)
C4	0.0737 (18)	0.087 (2)	0.0698 (18)	−0.0161 (18)	−0.0008 (17)	0.0115 (19)
C5	0.0827 (19)	0.073 (2)	0.0654 (17)	−0.0087 (18)	0.0111 (16)	−0.0025 (17)
C6	0.0586 (14)	0.0540 (15)	0.0634 (15)	−0.0009 (13)	0.0099 (12)	−0.0011 (13)
C7	0.0647 (14)	0.0334 (11)	0.0434 (10)	−0.0016 (11)	0.0072 (11)	−0.0012 (9)
C8	0.0702 (15)	0.0467 (15)	0.0509 (12)	−0.0030 (14)	0.0146 (12)	−0.0053 (11)
C9	0.084 (2)	0.0538 (18)	0.0657 (17)	−0.0167 (17)	0.0161 (17)	0.0060 (14)
C10	0.101 (2)	0.0387 (13)	0.0684 (17)	−0.0104 (15)	0.0068 (18)	0.0037 (13)
C11	0.094 (2)	0.0341 (13)	0.084 (2)	0.0069 (16)	0.0097 (19)	−0.0004 (14)
C12	0.0739 (18)	0.0386 (13)	0.0774 (19)	0.0019 (14)	0.0182 (15)	0.0022 (14)
C13	0.0657 (19)	0.079 (2)	0.109 (3)	0.023 (2)	0.0120 (19)	0.004 (2)
C14	0.118 (3)	0.141 (5)	0.080 (2)	−0.039 (4)	−0.019 (3)	0.003 (3)
S2	0.0532 (3)	0.0368 (3)	0.0479 (3)	0.0046 (2)	−0.0059 (2)	0.0046 (2)
O3	0.0603 (11)	0.0653 (13)	0.0561 (10)	0.0023 (10)	−0.0154 (9)	0.0038 (10)
O4	0.0918 (14)	0.0341 (9)	0.0735 (13)	0.0111 (10)	−0.0019 (11)	0.0091 (10)
N2	0.0620 (12)	0.0344 (10)	0.0564 (11)	0.0082 (10)	0.0091 (10)	−0.0013 (9)
C15	0.0500 (11)	0.0483 (12)	0.0505 (12)	−0.0020 (11)	−0.0022 (11)	0.0131 (11)
C16	0.0647 (17)	0.0704 (19)	0.0669 (17)	−0.0195 (16)	−0.0227 (15)	0.0204 (15)
C17	0.063 (2)	0.107 (3)	0.102 (3)	−0.021 (2)	−0.027 (2)	0.044 (3)
C18	0.0586 (17)	0.117 (4)	0.109 (3)	0.010 (2)	0.013 (2)	0.062 (3)
C19	0.073 (2)	0.097 (3)	0.086 (2)	0.021 (2)	0.0239 (19)	0.028 (2)
C20	0.0652 (17)	0.0663 (18)	0.0587 (15)	0.0104 (15)	0.0058 (12)	0.0105 (14)
C21	0.0425 (10)	0.0360 (12)	0.0569 (13)	0.0015 (10)	0.0081 (10)	0.0029 (10)
C22	0.0685 (16)	0.0403 (14)	0.0618 (14)	−0.0002 (13)	0.0056 (13)	−0.0040 (12)
C23	0.077 (2)	0.0372 (13)	0.089 (2)	−0.0024 (14)	0.0117 (18)	−0.0048 (15)
C24	0.0722 (18)	0.0446 (17)	0.089 (2)	−0.0011 (15)	0.0141 (18)	0.0130 (16)
C25	0.0773 (18)	0.0575 (19)	0.073 (2)	0.0020 (15)	0.0124 (17)	0.0208 (17)
C26	0.0635 (14)	0.0470 (15)	0.0559 (14)	0.0000 (13)	0.0099 (12)	0.0021 (12)
C27	0.125 (4)	0.095 (3)	0.084 (3)	−0.024 (3)	−0.038 (3)	−0.015 (2)
C28	0.065 (2)	0.177 (5)	0.167 (5)	0.019 (3)	0.018 (3)	0.071 (5)

### Geometric parameters (Å, °)

**Table:**

S1—O2	1.4278 (19)	S2—O3	1.429 (2)
S1—O1	1.433 (2)	S2—O4	1.435 (2)
S1—N1	1.632 (2)	S2—N2	1.619 (2)
S1—C1	1.760 (3)	S2—C15	1.758 (3)
N1—C7	1.426 (3)	N2—C21	1.421 (3)
N1—H1N	0.8600	N2—H2N	0.8600
C1—C6	1.379 (4)	C15—C20	1.386 (4)
C1—C2	1.404 (4)	C15—C16	1.391 (4)
C2—C3	1.373 (6)	C16—C17	1.421 (6)
C2—C13	1.517 (5)	C16—C27	1.501 (6)
C3—C4	1.384 (6)	C17—C18	1.366 (8)
C3—H3	0.9300	C17—H17	0.9300
C4—C5	1.380 (6)	C18—C19	1.354 (7)
C4—C14	1.502 (6)	C18—C28	1.520 (6)
C5—C6	1.373 (5)	C19—C20	1.379 (5)
C5—H5	0.9300	C19—H19	0.9300
C6—H6	0.9300	C20—H20	0.9300
C7—C12	1.374 (4)	C21—C26	1.372 (4)
C7—C8	1.377 (4)	C21—C22	1.390 (4)
C8—C9	1.390 (4)	C22—C23	1.382 (4)
C8—H8	0.9300	C22—H22	0.9300
C9—C10	1.377 (6)	C23—C24	1.367 (6)
C9—H9	0.9300	C23—H23	0.9300
C10—C11	1.347 (6)	C24—C25	1.381 (6)
C10—H10	0.9300	C24—H24	0.9300
C11—C12	1.382 (4)	C25—C26	1.375 (4)
C11—H11	0.9300	C25—H25	0.9300
C12—H12	0.9300	C26—H26	0.9300
C13—H13A	0.9600	C27—H27A	0.9600
C13—H13B	0.9600	C27—H27B	0.9600
C13—H13C	0.9600	C27—H27C	0.9600
C14—H14A	0.9600	C28—H28A	0.9600
C14—H14B	0.9600	C28—H28B	0.9600
C14—H14C	0.9600	C28—H28C	0.9600
			
O2—S1—O1	117.09 (14)	O3—S2—O4	117.71 (14)
O2—S1—N1	105.17 (14)	O3—S2—N2	109.64 (12)
O1—S1—N1	109.11 (13)	O4—S2—N2	104.73 (13)
O2—S1—C1	111.43 (15)	O3—S2—C15	106.81 (14)
O1—S1—C1	107.33 (13)	O4—S2—C15	110.97 (14)
N1—S1—C1	106.18 (12)	N2—S2—C15	106.48 (12)
C7—N1—S1	122.50 (18)	C21—N2—S2	125.67 (18)
C7—N1—H1N	118.7	C21—N2—H2N	117.2
S1—N1—H1N	118.7	S2—N2—H2N	117.2
C6—C1—C2	120.2 (3)	C20—C15—C16	120.5 (3)
C6—C1—S1	118.1 (2)	C20—C15—S2	118.0 (2)
C2—C1—S1	121.7 (2)	C16—C15—S2	121.5 (3)
C3—C2—C1	116.8 (3)	C15—C16—C17	115.6 (4)
C3—C2—C13	119.8 (3)	C15—C16—C27	123.8 (4)
C1—C2—C13	123.4 (3)	C17—C16—C27	120.6 (4)
C2—C3—C4	124.1 (3)	C18—C17—C16	123.8 (4)
C2—C3—H3	117.9	C18—C17—H17	118.1
C4—C3—H3	117.9	C16—C17—H17	118.1
C5—C4—C3	117.4 (4)	C19—C18—C17	118.4 (4)
C5—C4—C14	120.6 (4)	C19—C18—C28	121.1 (6)
C3—C4—C14	122.0 (4)	C17—C18—C28	120.5 (5)
C6—C5—C4	120.6 (4)	C18—C19—C20	120.9 (5)
C6—C5—H5	119.7	C18—C19—H19	119.6
C4—C5—H5	119.7	C20—C19—H19	119.6
C5—C6—C1	120.9 (3)	C19—C20—C15	120.8 (4)
C5—C6—H6	119.5	C19—C20—H20	119.6
C1—C6—H6	119.5	C15—C20—H20	119.6
C12—C7—C8	120.2 (3)	C26—C21—C22	120.0 (3)
C12—C7—N1	121.4 (3)	C26—C21—N2	118.9 (2)
C8—C7—N1	118.3 (2)	C22—C21—N2	121.0 (3)
C7—C8—C9	118.9 (3)	C23—C22—C21	118.8 (3)
C7—C8—H8	120.5	C23—C22—H22	120.6
C9—C8—H8	120.5	C21—C22—H22	120.6
C10—C9—C8	120.4 (3)	C24—C23—C22	121.3 (3)
C10—C9—H9	119.8	C24—C23—H23	119.3
C8—C9—H9	119.8	C22—C23—H23	119.3
C11—C10—C9	119.9 (3)	C23—C24—C25	119.4 (3)
C11—C10—H10	120.1	C23—C24—H24	120.3
C9—C10—H10	120.1	C25—C24—H24	120.3
C10—C11—C12	120.9 (3)	C26—C25—C24	120.1 (4)
C10—C11—H11	119.6	C26—C25—H25	120.0
C12—C11—H11	119.6	C24—C25—H25	120.0
C7—C12—C11	119.6 (3)	C21—C26—C25	120.4 (3)
C7—C12—H12	120.2	C21—C26—H26	119.8
C11—C12—H12	120.2	C25—C26—H26	119.8
C2—C13—H13A	109.5	C16—C27—H27A	109.5
C2—C13—H13B	109.5	C16—C27—H27B	109.5
H13A—C13—H13B	109.5	H27A—C27—H27B	109.5
C2—C13—H13C	109.5	C16—C27—H27C	109.5
H13A—C13—H13C	109.5	H27A—C27—H27C	109.5
H13B—C13—H13C	109.5	H27B—C27—H27C	109.5
C4—C14—H14A	109.5	C18—C28—H28A	109.5
C4—C14—H14B	109.5	C18—C28—H28B	109.5
H14A—C14—H14B	109.5	H28A—C28—H28B	109.5
C4—C14—H14C	109.5	C18—C28—H28C	109.5
H14A—C14—H14C	109.5	H28A—C28—H28C	109.5
H14B—C14—H14C	109.5	H28B—C28—H28C	109.5
			
O2—S1—N1—C7	−164.3 (2)	O3—S2—N2—C21	−67.5 (3)
O1—S1—N1—C7	69.3 (3)	O4—S2—N2—C21	165.3 (2)
C1—S1—N1—C7	−46.1 (3)	C15—S2—N2—C21	47.7 (3)
O2—S1—C1—C6	−133.9 (2)	O3—S2—C15—C20	5.2 (3)
O1—S1—C1—C6	−4.5 (3)	O4—S2—C15—C20	134.7 (2)
N1—S1—C1—C6	112.1 (2)	N2—S2—C15—C20	−111.9 (2)
O2—S1—C1—C2	47.0 (3)	O3—S2—C15—C16	−175.1 (2)
O1—S1—C1—C2	176.5 (2)	O4—S2—C15—C16	−45.6 (3)
N1—S1—C1—C2	−67.0 (3)	N2—S2—C15—C16	67.8 (3)
C6—C1—C2—C3	1.0 (4)	C20—C15—C16—C17	−0.5 (4)
S1—C1—C2—C3	−180.0 (3)	S2—C15—C16—C17	179.8 (2)
C6—C1—C2—C13	179.5 (3)	C20—C15—C16—C27	−180.0 (3)
S1—C1—C2—C13	−1.4 (4)	S2—C15—C16—C27	0.4 (5)
C1—C2—C3—C4	−1.0 (5)	C15—C16—C17—C18	0.4 (6)
C13—C2—C3—C4	−179.6 (4)	C27—C16—C17—C18	179.9 (4)
C2—C3—C4—C5	0.8 (6)	C16—C17—C18—C19	0.1 (6)
C2—C3—C4—C14	−178.8 (4)	C16—C17—C18—C28	178.7 (4)
C3—C4—C5—C6	−0.6 (5)	C17—C18—C19—C20	−0.6 (6)
C14—C4—C5—C6	179.0 (4)	C28—C18—C19—C20	−179.2 (4)
C4—C5—C6—C1	0.6 (5)	C18—C19—C20—C15	0.5 (6)
C2—C1—C6—C5	−0.8 (5)	C16—C15—C20—C19	0.1 (5)
S1—C1—C6—C5	−179.9 (3)	S2—C15—C20—C19	179.8 (3)
S1—N1—C7—C12	−45.2 (4)	S2—N2—C21—C26	−142.9 (2)
S1—N1—C7—C8	135.7 (2)	S2—N2—C21—C22	39.2 (4)
C12—C7—C8—C9	−2.4 (5)	C26—C21—C22—C23	0.5 (4)
N1—C7—C8—C9	176.7 (3)	N2—C21—C22—C23	178.4 (3)
C7—C8—C9—C10	2.2 (5)	C21—C22—C23—C24	−1.5 (6)
C8—C9—C10—C11	−0.5 (6)	C22—C23—C24—C25	1.0 (6)
C9—C10—C11—C12	−1.0 (6)	C23—C24—C25—C26	0.4 (6)
C8—C7—C12—C11	1.0 (5)	C22—C21—C26—C25	0.9 (4)
N1—C7—C12—C11	−178.1 (3)	N2—C21—C26—C25	−177.0 (3)
C10—C11—C12—C7	0.8 (6)	C24—C25—C26—C21	−1.3 (5)

### Hydrogen-bond geometry (Å, °)

**Table:**

*D*—H···*A*	*D*—H	H···*A*	*D*···*A*	*D*—H···*A*
N1—H1N···O3^i^	0.86	2.41	3.164 (3)	147
N2—H2N···O1^ii^	0.86	2.22	3.056 (3)	164
C11—H11···O2^iii^	0.93	2.50	3.227 (4)	135
C23—H23···O4^iii^	0.93	2.50	3.316 (4)	147

Symmetry codes: (i) −*x*+1, −*y*+1, *z*−1/2; (ii) *x*+1/2, −*y*+1, *z*; (iii) *x*, *y*+1, *z*.
